# Microsurgical and endovascular treatment of un-ruptured cerebral aneurysms by European hybrid neurosurgeons to balance surgical skills and medical staff management

**DOI:** 10.1007/s00701-021-04746-x

**Published:** 2021-02-10

**Authors:** Abdul Rahman Al-Schameri, Som Thakur, Michael Kral, Christoph Schwartz, Slaven Pikija, Camillo Sherif, Friedrich Weymayr, Bernd Richling

**Affiliations:** 1 Department of Neurosurgery, University Hospital Salzburg, Paracelsus Medical University, Salzburg, Austria; 2 Department of Neurology, Paracelsus Medical University, Salzburg, Austria; 3 Department of Neurosurgery, Karl Landsteiner University of Health Sciences, University Hospital St. Poelten, St. Poelten, Austria; 4 Department of Neuroradiology, Paracelsus Medical University, Salzburg, Austria; 5 Paracelsus Medical University, Salzburg, Austria

**Keywords:** Un-ruptured intracranial aneurysm, Microsurgery, Embolization, Hybrid neurosurgery, Outcome, Hospital economy

## Abstract

**Background:**

In Europe, aneurysm treatment performed by dually trained neurosurgeons is extremely scarce. We provide outcome data for un-ruptured aneurysm patients treated at a European hybrid center to prove that hybrid neurosurgeons achieve clinical and angiographical results allowing to integrate hybrid neurosurgery into routine aneurysm treatment. This will not only help to maintain neurovascular microsurgical skills but will influence staff costs in related hospitals.

**Methods:**

We retrospectively analyzed all consecutively treated un-ruptured aneurysm patients between 2000 and 2016. The decision-making took into account the pros and cons of both modalities and considered patient and aneurysm characteristics. Clinical outcome was assessed by the modified Rankin scale (mRS). Occlusion rates were stratified into grade I for 100%, grade II for 99–90%, and grade III for <90% occlusion. To account for the introduction of stents, two treatment periods (p1, 2000 to 2008; p2, 2009 to 2016) were defined.

**Results:**

The study population consisted of 274 patients (median age 55 years) harboring 338 un-ruptured aneurysms. Microsurgery (MS) was performed in 51.8% and endovascular therapy (EVT) in 43.1%; 5.1% required combined treatment. Overall, 93% showed a favorable clinical outcome (mRS 0–2), 94.3% after MS and 91.5% after EVT. Grade I aneurysm occlusion was achieved in 82.6% patients, 91.9% after MS and 72.9% after EVT. Procedure-related complications occurred after MS in 5.6% and after EVT in 4.4% patients. Mortality was recorded for five (1.8%) patients, one patient after MS and four after EVT. For the EVT cohort, significant improvement from p1 to p2 was seen with clinical outcomes (*P*=0.030, RR = 0.905, CI: 0.8351–0.9802) and occlusion rates (*P*=0.039, RR = 0.6790, CI: 0.499–0.923).

**Conclusion:**

Hybrid neurosurgeons achieve qualified clinical and angiographic results. Dual training will allow to maintain neurovascular caseloads and preserve future aneurysm treatment within neurosurgery. Furthermore economic benefits could be observed in hospital management.

## Introduction

Several studies have been published on the matter, whether one physician specialist is capable of performing both open microsurgical (MS) and neuroendovascular (EVT) techniques effectively and safely. However, almost all available data stem from non-European centers [4, 5]. In Europe, only de Vries [10] reported data from ruptured aneurysms treated by hybrid neurosurgeons at a single center.

In this retrospective study, we present clinical and angiographic long-term results of 274 patients harboring 338 un-ruptured aneurysms treated by dually trained neurosurgeons in a single European center in the period between July 2000 and December 2016, with the aim to compare these results to those from multi-modal teams or monotherapeutic centers and to evaluate whether results are sustainable or improve over time.

Based on the disclosed ability to perform dual aneurysm therapy efficiently and safely, we want to discuss the necessity to increase the number of “hybrid trained” neurosurgeons in Europe, with the aim to maintain neurovascular microsurgical skills [2, 10, 14] and with an eye to economize therapeutic manpower in related hospitals, this in the light of increasing numbers of endovascular therapy of cerebral stroke.

## Patients and methods

Blister and mycotic aneurysms were excluded from analysis. Of 285 patients diagnosed with un-ruptured aneurysms between 2000 and 2016, the clinical presentation was incidental in 29.2%, after previous SAH from another aneurysms in 20.8%, headache in 15%, ischemia in 12.8%, dizziness in 7.6%, seizures in 5.5%, and others (e.g., cranial nerve or brainstem compression) in 9.1%. Eleven patients were excluded: 3 with aneurysms less than 1.5 mm, 4 with fusiform aneurysms, 1 patient due to significant comorbidity, and three patients refused treatment. The clinical data of 274 patients and their 338 un-ruptured cerebral aneurysms were retrospectively reviewed and collected in a patient database. Demographic data, clinical baseline characteristics, aneurysm location, size, geometry, type of treatment or re-treatment, and the results and complications were extracted (Table 1). All complications were recorded as general or procedure related, with or without neurological consequences, and with transient or permanent neurological morbidity. According to governmental guidelines, no ethics committee approval was required for this study. A total of 221 patients harbored single aneurysms, and 53 patients had multiple aneurysms (*n*= 117). All aneurysms were saccular; 30 of them were giants*.* To illuminate the influence of intracranial stents, the observation time was divided into two periods; period 1 (p1) from 2000 to 2008 before and period 2 (p2) from 2009 to 2016 after the introduction of stents. The distribution of patients, aneurysms, periods, and their modalities are disclosed in (Table 2). A total of 260 (94.9%) patients underwent single mode treatment (Table 3), and 14 (5.1%) patients underwent multiple modality treatment. Crossover occurred in 5 patients of equivalence, 3 MCA aneurysm, and 2 ACOMA, where the wish of the patient was respected. Three patients refused the proposed treatment mode after comprehensive explanation; no treatment was done.

**Table 1:** Demographic data and baseline characteristics

	All	MS	EVT	Comb. modalities	P
Aneurysms	338	180	158	24	-
Patients	274	142	118	14	-
Age, median (IQR)	54.7 (47.7–61.1)	54.7 (47.7–61.3)	55.1 (48.9–62.9)	40.5 (45–36)	0.189
≤ 50 years	89 (32.5)	43 (52.8)	35 (43.8)	11 (3.4)	-
>50 and ≤ 70 years	154 (56.2)	83 (50.6)	68 (48.7)	3 (0.6)	-
> 70 years	31 (11.3)	16 (54.8)	15 (45.2)	0	-
Gender					0.391
Male	73 (26.6)	36 (49.3)	34 (46.6)	3	
Female	201 (73.4)	106(52.7)	84 (46.8)	11	
Previous SAH	71 (21.0)	40 (56.3)	31 (43.7)	8	0.086
Location					<0.001
ICA	83 (24.6)	13 (7.3)	70 (43.8)	7	
AcomA	53 (15.7)	31 (17.6)	22 (14.4)	2	
Pericallosal artery	10 (2.9)	8 (4.8)	2 (1.4)	0	
MCA	138 (40.8)	119 (64.8)	19(12.3)	11	
Pcom artery	17 (5.0)	3(1.8)	14 (7.5)	3	
Posterior circulation	37 (16.5)	6 (6.4)	31 (20.5)	1	
Size in mm* (*N*=338)					0.018
≤ 5 mm	119 (35.2)	70 (58.8)	49 (41.2)	7	
> 5 and ≤ 10 mm	166 (49.1)	92 (55.4)	74 (44.6)	12	
> 10 and ≤ 15 mm	23 (6.8)	11 (47.8)	12 (52.2)	3	
> 15 and > 25 mm	30 (8.9)	7 (23.3)	23 (76.7)	2	
Neck size					0.138
<= 4 mm	80 (23.7)	49 (61.2)	31 (38.8)	8	
> 4 mm	258 (76.3)	131 (50.8)	127 (49.2)	14	
Aspect ratio (*N*=258)	1.27 (1.00-1.73)	1.16 (0.91-1.50)	1.45 (1.16-1.88)		<0.001

*MS* microsurgery, *EVT* endovascular treatment, *AN* aneurysms, *IQR* interquartile range; T* treatment, *SAH* subarachnoid bleeding history, *ICA* internal carotid artery, *AcomA* anterior communicating artery, *MCA* middle cerebral artery, *VB* posterior circulation (vertebral, basilar, posterior inferior cerebellar, superior cerebellar)

*Largest size is calculated for neck, dome, or height of the aneurysm and the largest of three reported; aspect ratio is height/neck of the aneurysm

**Table 2:** Aneurysm locations, stratified to treatment modality and period of treatment

	AcomA	ICA	MCA	Pericall.A	PcomA	BA	PCA	SCA	Vert.A	Total
MS/p1	17	10	57	4	1	2	0	3	0	94
EVT/p1	12	36	10	1	8	12	1	2	0	82
MS/p2	14	3	62	4	2	1	0	0	0	86
EVT/p2	10	34	9	1	6	11	3	1	1	76
Total p1 Total p2	29 24	46 37	67 71	5 5	9 8	14 12	1 3	5 1	0 1	176 162
Total	53	83	138	10	17	26	4	6	1	338

*MS* microsurgery, *EVT* endovascular treatment, *P* period, *AcomA* anterior communicating artery, *ICA* internal carotid artery, *MCA* middle cerebral artery, *Pericall.A* pericallosal artery, *PcomA* posterior communicating artery, *BA* basilar artery, *PCA* posterior cerebral artery, *SCA* superior cerebellar artery, *Vert.* vertebral artery

**Table 3:** Clinical outcome (mRS) of patients after treatment by single or dual modalities and of patients with history of prior SAH, stratified to p1 and p2

a							
mRS	Single mode treatment (*n*=260, evaluated 258)					Total	
	p1 (*n*=133)			p2 (*n*=125)			
	MS (*n*=70)		EVT (*n*=63)	MS (*n*=71)	EVT (*n*=54)	*n*=258	
mRS (0–2)	63 (90%)		55 (87.3%)	70 (98.6%)	52 (96.3%)	240 (93.0%)	
0	51		40	56	43		
1	11		14	11	8		
2	1		1	3	1		
mRS (3–5)	6		6	1	0	13 (5.0%)	
3	4		0	1	0		
4	2		4	0	0		
5	0		2	0	0		
Death	1		2	0	2	5 (1. 9%)	
b							
mRS	Multiple mode treatment (*n*=14)					Total	
	p1 (*n*=9)			p2 (*n*=5)		*n*=14	
mRS (0–2)	9(100%)			5(100%)		14(100)	
0	2			2			
1	7			3			
2	0			0			
mRS (3–5)	0			0		0(%)	
3	0			0			
4	0			0			
5	0			0			
Death	0			0		0(%)	
c							
		Patients post SAH (*n*=57, evaluated 56)					Total
mRS (0–6)	p1 (*n*=30)			p2 (*n*=26)			
	MS (*n*=15)	EVT (*n*=13)	MS+EVT (*n*=2)	MS (*n*=16)	EVT (n=9)	MS+EVT (*n*=1)	*n*=56
mRS (0–2)	12 (80%)	11 (84.6%)	2 (100%)	16 (100%)	8 (88.9%)	1 (100%)	50 (89.3%)
0	8	6	0	8	7	1	
1	3	5	2	8	1	0	
2	1	0	0	0	0	0	
mRS (3–5)	3 (20%)	1 (7.7%)	0	0	1 (11.1%)	0	5 (8.9%)
3	3	0	0	0	1	0	
4	0	1	0	0	0	0	
5	0	0	0	0	0	0	
Death	0	1 (7.7%)	0	0	0	0	1 (1.8%)

*mRS* modified Rankin scale, favorable outcome (0–2), moderate or severe disability (3–5)

The decision to treat small- and mid-sized UIAs followed published standards [7, 21, 28]; the indication to treat aneurysms smaller than 5.0 mm was based on the presence of at least two of the following criteria: female sex together with age 40–60 years, cigarette smoking, family history (first-degree relatives with history of SAH), posterior location *,* multilobulated shape, and aneurysm growth.

The decision-making between endovascular and microsurgical treatment was primary based on a consideration of the advantages and disadvantages of the two treatment tools and their influence on the specific outcome of the patient, e.g., the ability of clips to reconstruct vascular structures, versus the sometimes limited stability of the endovascular aneurysm occlusion (later on improved by the introduction of stents) and the amount of invasivity of the surgical versus the endovascular approach. In addition, aneurysm factors like location, size, shape and architecture, endo-aneurysmal thrombus, wall calcification, adhesion or obliteration of the Sylvain fissure, blood flow conditions, and relationship to surrounding vessels were taken to account. The further indication workflow includes general factors, such as patient age, comorbidities, and potential contraindication for dual platelet anticoagulation after a deployment of stents.

The resulting therapeutic decision was hence tailored specifically to each patient and each specific aneurysm, with a basic attempt to avoid tool-specific disadvantages and to gain tool-specific advantages. In some patients, however, a combination of both modalities has become necessary. The resulting arguments for each aneurysm treatment were explained to each patient, including information about the specific natural risk of the disease, the aneurysm’s morphology, and the risks inherent in the therapeutic options. Finally, a consent for the chosen therapeutic approach was achieved.

### Therapeutic methods

All treatments were performed by 3 board-certified neurosurgeons (BR, AA, MK). The senior author BR started his EVT experience 1982 under the supervision of Luc Picard/Nancy and thereafter established EVT at the Neurosurgical University Clinic Vienna, Austria. The first author AA started his training in 1994 at Vienna EVT center and subsequently continued performing EVT in Vienna and after 1998 in Salzburg; he is an accredited “Fellow of the European Board of Neurointervention.” Co-author MK had started his Neuroendovascular training in Salzburg 2010 and is meanwhile an experienced hybrid neurosurgeon with an annual endovascular case load of 40–50 patients.

All surgical procedures were performed with standard microsurgical techniques; temporary clipping was applied in 8 cases. Endovascular therapy consisted in p1 of coiling only, in p2 of the addition of permanent stent-assisted coiling, and the use of flow-diverting stents.

In patients with very large or giant aneurysms, our primary concept consists of an extra-intracranial bypass to the aneurysm-bearing territory, followed by an endovascular test occlusion of the aneurysm-supplying artery, and finally surgical aneurysm trapping.

### Clinical and radiological data*,* statistical analysis

Clinical outcome-related data were recorded according to the modified Rankin scale (mRS) [38]. The surgical group was followed clinically at discharge and at further controls in the outpatient clinic for a median period of 28 months (IQR −93), the endovascular group at discharge and for a median of 32 months (IQR 8–75). For the study, the last clinical and neurological follow-up was taken for analysis. The preoperative mRS score was (0) in 190 (69.3%) patients, (1) in 76 (27.7%) patients, (2) in 4 (1.5%) patients, (3) in 2 (0.7%) patients, and (4) in 2 (0.7%) patients. The latter 4 patients had suffered from prior SAH from another aneurysm.

For radiological evaluation, MS-treated patients underwent 4-vessel angiography 6–12 months after surgery for a median of 15 months (IQR 6–53). Patients after EVT underwent MRA (TOF) at the first postoperative day and 3–6–12 months for a median of 33 months (IQR 9–76), with subsequent individualized MRA examinations in case of remnants. If necessary, patients were scheduled for angiography with intention to retreat. Occlusion rates were classified according to the Raymond scale [32] (grade 1, complete obliteration = 100%; grade 2, residual neck = 99.9–90%, grade 3, residual aneurysm = <90%). To estimate angiographic occlusion, for the surgical group, a modified version of the classification proposed by Sindou [1, 34] was used: grade 1 was added representing 100% occlusion, grade 2 = 99.9–90%, and grade 3 = 89.9–70%.

For statistical analysis, the patient demographics, clinical data, and radiological parameters were analyzed by means of descriptive statistics. Since our interval variables were non-normally distributed, we performed a Kruskal-Wallis non-parametric test for differentiation between groups. Fisher’s exact test was performed for categorical variables. For statistical evaluation, clinical outcomes were stratified as favorable with an mRS of 0–2 versus poor with an mRS of 3–6. Multivariate associations between different variables were analyzed using logistic regression with binary outcomes to report the odds ratio. First, we limited the observations to patients harboring aneurysms who were treated with one technique, thus excluding three patients with four UIAs treated with both surgical and endovascular techniques. Of these 252 patients, 249 patients had an available outcome at 3 months and were considered for further analysis. Statistical significance was set to be positive below 0.05. Statistical analysis was performed using STATA 13.0 (StataCorp LLC, TX).

## Results

### Clinical results

Two/274 patients were lost for long-term follow-up. There was only one bleeding; this patient bled 1 day after EVT. Excellent or good clinical outcomes could be achieved after MS in p1/p2 in 63 (90%)/70 (98.6%), after EVT in 55 (87.3%)/52 (96.3%). Morbidity was in p1/p2 6/1 after MS and 6/0 after EVT. The clinical improvements seen from p1 to p2 concerning reduced morbidity showed significance only for EVT (*P*=0.030, RR = 0.905, CI: 0.8351–0.9802). Overall mortality was seen in 5 (1.8%), 1 (0.7%) in the surgical (p1), and 4 (3.4%) in the EVT group (2 p1/2 p2). The surgical patient died after rupture during clipping; from the four embolized patients, 2 had giant aneurysm (one intraprocedural rupture by guidewire and one parent vessel occlusion after stent and coils), and one patient died after stent and coils due to an ICH of unknown origin (non-ischemic). The fifth patient died from an acute SDH of unclear mechanism after a stent/coil procedure on a large basilar tip aneurysm. Treatment by both modalities (MS after EVT, EVT after MS) was applied to 14 (5.1%) patients. All those patients achieved a favorable clinical outcome, nine in p1 and 5 in p2 (Table 3). Complications with permanent neurological deficits occurred in 10 (5.6%) patients of the MS group and 7 (4.4%) in the EVT group, stratified to p1/p2, after MS in 5.5%/2.2% and after EVT in 2.2%/0.4% (Table 6). Of the 4 patients treated with mRS grade> 2, 2 patients remained unchanged, and 2 patients showed a functional improvement compared to prior their respective preoperative status.

### Angiographic results

After MS, total occlusion was seen in 91.9%, after EVT in 72.9%. Stratified to periods, no difference was seen after MS, but after EVT, the rate of aneurysm residuals decreased significantly from 20 to 9 (*P*=0.039, RR = 0.6790, CI: 0.499–0.923) (Table 4 section a). Occlusion in large and giant aneurysms (Table 5) was 100% in all 7 surgical cases, all treated with bypass and aneurysm trapping. After EVT, 17/23 showed grade I, 2 grade II, and 4 grade III (all 6 latter in p1). Of 24 patients retreated (20 repeated EVT, 4 combined modes), 23 were followed, 19 showing total occlusion.

**Table 4:** Finale angiographic outcome of 338 aneurysms, thereof 24 aneurysms after retreatment, stratified to treatment modes and periods; and angiographic outcome immediately post-procedure and at last follow up of aneurysms treated with coils and stent-assisted coiling

a					
Angiographic OCR*	Total aneurysms (*n*=338, evaluated= 316)				Total
	p1 (*n*=156)		p2 (*n*=160)		316
	MS (*n*=77)	EVT (*n*=79)	MS (*n*=84)	EVT (*n*=76)	161/155
I	73 (94, 8%)	53 (67, 1%)	75 (89, 3%)	60 (78, 9%)	261(82, 6%)
II	0	6	2	7	15
III	4	20	7	9	40
b					
Angiographic OCR*	Aneurysms (*n*=24) after 43 retreatments, evaluated= 23)				
	p1 (*n*=17)		p2 (*n*=6)		
	EVT (*n*=15)	MS+EVT (*n*=2)	EVT (*n*=4)	MS+EVT, EVT+MS (*n*=2)	
I	12 (80%)	2 (100%)	3 (75%)	2 (100%)	
II	0	0	1	0	
III	3	0	0	0	
c					
Angiographic OCR*	Angiography after EVT with coils (*n*=99)				
	Post-procedure		At follow up		
I	78 (83.0 %)		68 (70.8%)		
II	11		9		
III	5		19		
**)	5		3		
	99		96 ***)		
d					
Angiographic OCR*	Angiography after EVT with stent+ coils (*n*=59)				
	Post-procedure		At follow up		
I	28 (50%)		45 (76.3%)		
II	18		4		
III	10		10		
**)	3		0		
	59		59		

*OCR occlusion rate, *MS* microsurgery, *EVT* endovascular treatment

**) 8 patients post procedure angio-protocol lost

***) 3 patients follow up angio-protocol lost

**Table 5:** Angiographic outcome of 317 aneurysms (on 256 patients), stratified by size and treatment period

Aneurysm size <5mm (*n*=113)				
	p1 (*n*=47)		p2 (*n*=66)	
OCR*/modality	MS (*n*=29)	EVT (*n*=18)	MS (*n*=37)	EVT (*n*=29)
I	28 (96.6%)	13 (72.2%)	34 (91.9%)	24 (82.8%)
II	0	0	1	1
III	1	5	2	4
Aneurysm size 5–10mm (*n*=153)				
	p1 (*n*=82)		p2 (*n*=71)	
OCR*/modality	MS (*n*=40)	EVT (*n*=42)	MS (*n*=40)	EVT (*n*=31)
I	37 (92.5%)	29 (69%)	35 (87.5%)	23 (74.2%)
II	0	4	1	4
III	3	9	4	4
Aneurysm size 10–15mm (*n*=21)				
	p1 (*n*=10)		p2 (*n*=11)	
OCR*/modality	MS (*n*=4)	EVT (*n*=6)	MS *(n*=3)	EVT (*n*=8)
I	4 (100%)	4 (66.7%)	2 (66.7%)	7 (87.5%)
II	0	0	0	1
III	0	2	1	0
Aneurysm size >15mm (*n*=30)				
	p1 (*n*=18)		p2 (*n*=12)	
OCR*/modality	MS (*n*=3)	EVT (*n*=15)	MS (*n*=4)	EVT (*n*=8)
I	3 (100%)	9 (60%)	4 (100%)	8 (100%)
II	0	2	0	0
III	0	4	0	0

*OCR occlusion rate

## Discussion

### Organizational background

The development of neuro-endovascular therapies has led to three models of task sharing. In some countries, dedicated centers developed for endovascular therapy while other subspecialized in microsurgery [8, 23, 27, 36]. Other countries formed multidisciplinary teams with subspecialized micro- and endovascular surgeons, who discuss cases together and recommend therapies based on consensus on best benefit for individual patients [9, 18, 20, 30]. In this model, the patient can be offered both treatment modalities in one center, but the balance of decision-making will depend on the equivalence of the team members. Mutual knowledge about advantages and disadvantages of all therapeutic options is mandatory, but even more important is practical experience of the responsible therapists in both methods.

A third model is the single-surgeon dual competence model, the hybrid neurosurgeon. This model is certainly less costly, covering both treatments in one person, including eventually interventional stroke therapy. Decisions by dually trained neurosurgeons shall follow reproducible pattern (see above), but the hybrid neurosurgeon model has been criticized, suggesting that one person may not be able to keep sufficient expertise for both modalities, and that the model would thereby prove to be less acceptable or even cost-effective than the multi-modal team model. In the meantime, numerous publications from non-European centers could prove the equivalence of hybrid neurosurgeons [2–4, 10, 13, 15, 19, 33, 35, 39, 40].

In Europe, however, only one study has been published so far, disclosing the equivalence of hybrid neurosurgeons therapy, but only for ruptured brain aneurysms [10]. This reflects the current European situation, where due to the rapid and successful development of neuro-endovascular therapies, a continuous shift of indications for brain aneurysm treatment towards endovascular concepts occurred. This development is potentially problematic for a subgroup of patients that cannot be treated by endovascular means and would need microsurgery (very small but bleeding aneurysms, MCA-aneurysms with unfavorable geometry, aneurysms with intraparenchymal hematoma, giant and partial thrombosed aneurysms, young patients who may be noncompliant with follow-up imaging, high-grade SAB) and also may endanger the maintenance of neurovascular microsurgical skills.

### Indication and decision-making

Especially in un-ruptured aneurysms, the indication to treat, and if yes, by which therapeutic mode, will be crucial for the result. The therapeutic decision will positively be influenced by the hybrids capacity to assess and compare the advantages and disadvantages of both therapeutic options in detail, avoiding wrong decisions because the “availability of just a hammer lets look all targets like nails”. To allow for the inclusion of all influencing factors, a comprehensive registration of data is mandatory, taking into account parameters from “both sides,” like, e.g., the aspect of the Sylvian fissure in MRI (surgical approach to M1/M2 bifurcation) and the tolerance for antiplatelet therapy (stent deployment into M1/M2). The patients therapeutic expectation can be influenced by extensive explications; our number of “refusals” was extremely low (8/274); only 3/8 patients did finally not undergo the proposed therapy.

### Results

This is to our knowledge the first European report about hybrid neurosurgical therapy for un-ruptured brain aneurysms, with the aim to prove the equivalence of European hybrid neurosurgeons on a non SAH aneurysm patient cohort. Comparing first our clinical outcome data to those from monotherapeutic centers, we looked at King [17] who analyzed in a meta-analysis the outcome data from 38 studies after surgery for UIA’s, disclosing a general mortality of 1% and general morbidity of 4.1%, or at Raaymakers [29] who analyzed 61 studies on surgery for UIAs disclosing a general mortality of 2.6% and a morbidity of 10.9% and saw our clinical results in good order. For endovascular UIA therapy, there is recent information given by Kallmes [16] who described in an international retrospective multicenter study mortality after stent embolizations as 3.8% and with giant aneurysms as 10.9%.and others [6, 22]. Although the capacity of microsurgery to reconstruct vessel walls is known to be higher than from endovascular tools, publications indicate that there is a significant rate of incomplete aneurysm occlusions even after surgical treatment (Dellaretti as 10.2%, Nussbaum as 8%, and Nanda as 6% [11, 23, 24]). Our surgical occlusion rate of 92% is well comparable to these data. Recent and well comparable angiographic endovascular results were given by Pierot [26]. His rate of totally occluded un-ruptured aneurysms after single endovascular treatment was 63% (in our material 72.9%) with neck remnants in 22.5% (8.4%) and aneurysm remnants in 14.6% (18.7%) aneurysms. Comparing our outcome data to those from hybrid centers, we looked at the work of Bekelis [4], who reported for a series of un-ruptured aneurysms treated by hybrid neurosurgeons an overall 1-year mortality of 5.0%, or Alexander [2], who disclosed a mortality after UIA therapy of 0.85%. Our overall mortality (1.8%) and the surgical value (0.7%) were satisfying; the endovascular mortality was 3.4% due to two giant aneurysms.

### Periods

Comparing the two time periods p1 and p2, the following information can be taken: The significant p1/p2 improvement of aneurysm occlusion with stent supported EVT (Table 4) could be seen in conjunction with the significantly improved clinical results in p2 (Table 3) due to the elegance of stent deployment versus increased efforts in p1 to pack coils as dense as possible, necessary to prevent from aneurysm reperfusion. Comparing occlusion rates at the end of the (last) procedure(s) to the last follow -up angiography (Raymond I+II versus III), we see in the group of coiled aneurysms a significant decrease in total occluded aneurysms from 78 (83.0%) to 68 (70.8%), (*P*=0.0038, RR = 2.573, CI: 1.165–5.686). Otherwise, in the group of 59 stented aneurysms (49 stent-assisted coiling procedures and 10 flow-diverting stents), we found a slight increase in total occlusions from 28 (50%) to 45 (76.3%), showing good aneurysm stability (Table 4 sections c and d).

The p1/p2 decrease in complications, especially with EVT, can be seen as a learning effect, even if statistically not significant (Table 6).

**Table 6:** Complications

General complications -Major complication (*n*=3), pulmonary embolism, pneumothorax, cardiac arrhythmia -Minor complication (*n*=6), exanthema, otitis, periodontitis, electrolyte disturb	*n*=9 (3.3%) MS *n*=2, EVT=1, MS *n*=2, EVT *n*=4		
Stratified to periods and modalities	P1 (*n*=5) 1.8% MS *n*=2/EVT *n*=3	P2 (*n*=4) 1.5% MS *n*=2/EVT *n*=2	
Procedure-related complications, general	P1 *n*=29 (10.6%)	*n*=61 (22.3%)	P2 *n*=32 (11.6%)
Stratified to modalities	MS *n*=42, EVT *n*=19		
No-neurological consequences -CSF leak, retroperitoneal/groin hematoma, wound dehiscence	MS *n*=8, EVT *n*=6		
Neurological consequences, general ***-***Major/minor ischemic complications, ICH,EDH, SDH, seizure, chronic, headache, visual disturbance	*n*=47		
Neurological permanent clinical consequences	*n*=17		
-Stratified to periods and modalities	P1	P2	
MS	4 (5.5%)	6 (2.2%)	
EVT	6 (2.2%)	1 (0.4%)	

*MS* microsurgery, *EVT* endovascular treatment, *ICH* intracerebral hematoma, *EDH* epidural hematoma, *SDH* subdural hematoma

### The hybrid model

The hybrid model allows for more than one advantage: first, the one-person principle allows for an indication making independent of organizational or local political influences; second, personally tailored and balanced indications lead to an appropriate selection of tools; and third, continuous practice of neurovascular surgery will guarantee for preservation of microsurgical skills, essential for vascular neurosurgery in general. As a matter of principle, these elements can be gained by a well-functioning multidisciplinary team as well, but such teams are scarce; their functionality is depending on to many influencing factors. Otherwise, qualified training in vascular neurosurgery followed by an substantial endovascular training [12, 13, 25, 31, 37] will be time-consuming, but the result will allow for a comprehensive understanding of cerebrovascular diseases and their therapeutic options.

### Limitations

The limitations of the present study reflect its purpose, since it reports retrospectively and shows monocentric design. Furthermore, there are indications and selection biases caused by the non-randomized treatment selection principles. This bias have influenced the statistical power but displayed the reality of a highly complex disease. Thus, these limitations are unavoidable in light of our effort to apply the most suitable therapeutic techniques to each particular patient and aneurysm.

## Conclusion

In contrast to the majority of the US or Asian countries, in Europe only a handful of vascular neurosurgeons works under “hybrid conditions”; at present the vast majority of neuro-endovascular procedures is performed by neuroradiologists. The consequence of this development is that in Europe the number of microsurgical aneurysm procedures decreases, and experience and skills in aneurysm surgery and cerebrovascular procedures in general are at risk. The presented indication concept and outcome data for the treatment of un-ruptured brain aneurysms performed by European neurosurgeons, experienced equally in surgical and endovascular techniques, are well comparable to those reported by monotherapeutic and hybrid centers. They prove, that also in Europe, dually trained neurosurgeons would be able to perform surgical and/or endovascular procedures effectively and safely. Balanced and specifically tailored indication concepts, the maintenance of vascular microsurgical skills, and furthermore, the availability of both therapies in the person of one single operator as a significant economic factor in hospital management are the benefits.

## Data Availability

All study data are included in the manuscript and supplementary material.
